# Epidemiology of Viral Hepatitis and Liver Diseases in Bangladesh

**DOI:** 10.5005/jp-journals-10018-1124

**Published:** 2015-01-06

**Authors:** Mamun-Al Mahtab

**Affiliations:** Department of Hepatology, Bangabandhu Sheikh Mujib Medical University, Shahbag, Dhaka, Bangladesh

**Keywords:** Epidemiology, Viral hepatitis, Liver disease, Bangladesh.

## Abstract

Bangladesh is a South Asian country with a homogeneous population. However, some difference has been demonstrated in the distribution of liver disease in different parts of the country. Hepatitis E remains the principal cause of acute hepatitis in Bangladesh, while chronic liver disease in Bangladesh, including hepatocelullar carcinoma, is predominantly due to hepatitis B virus. Hepatitis B has been extensively studied and characterized in Bangladesh, and some major clinical trials with HBV-related antigens have also been conducted.

**How to cite this article:**

Al-Mahtab M. Epidemiology of Viral Hepatitis and Liver Diseases in Bangladesh. Euroasian J Hepato-Gastroenterol 2015;5(1):26-29.

## INTRODUCTION

Bangladesh is located in South Asia with India on three sides and Bay of Bengal on the other and also sharing a small land border with Myanmar. It is one of the most densely populated countries of the world with a population of over 160 million in an area of 15,5000 square kilometers only. The population is very homogeneous with more than 95% being ethnic Bengalis. Liver diseases are major burden to the country’s health and interestingly, despite the relatively small area and a homogeneous population divergence in the prevalence of different liver diseases is seen across Bangladesh.

## HEPATITIS B

Bangladesh is in the intermediate prevalence zone of hepatitis B virus (HBV), with a 40% lifetime risk of acquiring the virus by its residents. Hepatitis B has been extensively studied in Bangladesh. The prevalence of HBV in Bangladesh is 5.4% with neonatal, perinatal, and preschool transmission, injection, treatment from quack, mass vaccination against chickenpox and cholera, hair cut and shaving at barber shop have been identified as the principal modes of transmission of the virus in the country.^1^ In a local study, liver biopsy was done in 141 inactive HBV carriers with no liver-related complains in order to evaluate the extent of hepatic inflammation and fibrosis. Although the patients were inactive HBV carriers, mild to moderate levels of necroinflammation (HAI-NI>7) was seen in 26% patients. Seventeen had severe hepatic fibrosis (HAI-F>3). A total of 10 patients had both moderate hepatic inflammation (HAI-NI>7) and sever hepatic fibrosis (HAI-F>3).^2^ Subsequently a much larger study evaluated 702 chronic HBV carriers detected incidentally. This later study exhibited low HBV DNA levels (>10^5^ copies/ml) in 49.3% and normal about 50% (ULN; >42 U/l). In spite of having low HBV DNA and normal ALT, significant levels of patients had moderate hepatic inflammation (HAI-NI≥7) and severe hepatic fibrosis (HAI-F≥3).^3^ Characteristics of chronic hepatitis B (CHB) have also been evaluated. Initially, the predominant genotype of HBV in Bangladesh was shown to be D (50%) followed by C (39%). Recently, we are analyzing about 500 randomly collected sera for genotyping and it appears that HBV genotype C is the prominent genotype of HBV in Bangladesh. As expected, genotype C is more frequently associated with raised ALT (94 *vs* 50%) and more extensive hepatic necroinflammation (64.7 *vs* 36.4%) in Bangladesh.^4^ Another study was conducted with 155 CHB patients to compare between HBeAg positive and negative infection. It was seen that although there was no significant difference in hepatic necroinflammation between these two groups, HBeAg negative CHB was associated with more advanced hepatic fibrosis (28.3 *vs* 19.6%).^5^ On the other hand, another study involving 159 CHB patients failed to reveal any correlation between severity of hepatic histo-pathological changes and HBV DNA level in both HBeAg positive and negative CHB.^6^ In a more recent study, the utility of ALT levels in predicting liver disease severity has been assessed. This study of 255 CHB patients clearly demonstrated that the levels of ALT is not indicative of liver damage in many cases.^7^

Economic burden of HBV on our economy has also been studied. It was calculated that if only 1°% of our HBV infected population receives treatment, it translates into US$ 16-1440 million per-annum.^8^

Several clinical trials have been conducted in Bangladesh, like combination therapy with lamivudine and vaccine in CHB^9^ and interferon and lamivudine in pediatric patients with CHB,^10^ which showed limited success and the more recent study of combination therapy with half dose and shorter duration pegylated interferon in combination with entecavir in CHB, which was very promising.^11^ However, the study currently ongoing in Bangladesh, that deserves special mention, is the phase III clinical trial involving a therapeutic vaccine (NASVAC) head to head with pegylated interferon in 160 CHB patients. Data emerging from the already completed phase I/II as well as this ongoing study suggests that NASVAC is likely to be as effective, but with much superior safety profile compared to the only other immune-modulator against HBV currently in the market, i.e. pegylated interferon.^12,13^

## HEPATITIS C

Several studies have been carried out in Bangladesh to assess the prevalence of HCV in different population. However, data vary considerably among regions and groups. In fact, in the absence of a properly designed population based study the exact HCV prevalence in Bangladesh remains a matter of debate. In one of the earliest publications in the 1990s, Khan et al reported zero prevalence of HCV in the country!^14^ This was, however, contradicted by Akbar et al who found more than 5% prevalence of HCV in apparently healthy Bangladeshis.^15^ Two decades later, in 2011, Mahtab et al reported 0.88% prevalence of the virus in adult residents of a locality adjacent to the capital city Dhaka.^16^ However, at around the same time, a study by Ashraf et al revealed a much lower prevalence of HCV at only 0.2% among the residents of an urban slum in Dhaka.^17^ Outside Dhaka city, Rudra et al found 6.25% prevalence of HCV in Mymensingh city of the country in 2011^18^ and a year earlier the same author found the prevalence of HCV to be 0.04% in Khulna city. Both these studies were carried out among blood donors in the transfusion medicine departments of two different public medical colleges in these two cities respectively. The prevalence of HCV in Bangladeshi immigrants to Europe is also found in the published literature. Aliberch et al reports 0.09% prevalence of the virus among young Bangladeshi immigrants to Spain,^19^ while in a study by Uddin et al, the figure is higher at 0.6% among Bangladeshi immigrants to UK, which has the largest Bangladeshi immigrant population in Europe.^20^ As expected high-risk population have a much higher prevalence of HCV in Bangladesh as elsewhere in the world. Shirin et al found the prevalence of HCV to be 24.8 and 5.8% respectively among intravenous and non-intravenous drug abusers.^21^ Interestingly, this figure has been shown to be only 2% among patients with beta thalassemia major.^21^

In the work by Mahtab et al the major risk factors identified for HCV infection in Bangladesh were treatment by unqualified and traditional practitioners, history of mass vaccination against smallpox, hair cutting and shaving by barbers and body piercing.^16^ Chakrabarty et al identified lack of people’s knowledge about transfusion transmitted infections as a risk factor for HCV transmission.^22^ The prevalence of HCV infection was significantly higher among the intravenous drug abusers in a study by Shirin et al, but the study did not find any association between transmission of HCV and sexual promiscuity.^21^ Waheed et al has observed that in developing countries like Bangladesh, nonimplementation of international standards regarding blood transfusion, shaving from barbers, reuse of needles for ear and nose piercing, reuse of injections, injecting drug users, tattooing, unsterilized dental and surgical instruments are important sources of HCV transmission.^23^ In fact, road side barber shaving has been reported to be very high (34-49%) in Bangladesh.^24^

According to a study by Mahtab et al genotype 3 is the predominant genotype of HCV in the country.^25^ The study revealed that 41% had genotype 3, 31°% had mixed genotypes 3 + 4 and 21%% had genotype 1. Patients also had genotypes 2, 4, 5 and mixed genotypes 5 + 6, the figure being 1.6% in each case. The same group, in another publication, also reported higher prevalence of genotype 3 (89.2%) in Bangladeshi HCV infected patients. In this paper, they also found 8.1% genotype 1 and 2.7% mixed genotypes 5 + 6.^26^

There are several papers reporting treatment response in HCV infected Bangladeshis with standard combination regimen of pegylated interferon and ribavirin. In a recent study Rahman et al, it is observed that genotype 3 (a, b) patients attained 47.05% sustained virological response (SVR) as opposed to 100% SVR attained in genotypes 1 (a, b), 3 and 4 mixed, 2b and 4 HCV infections.^27^ The same group observed late relapse of HCV in 4 of 52 patients with initial SVR over a 5 years follow-up period. Relapse was more common in patients with cirrhosis of liver (50%) against (2.17%) without cirrhosis.^28^ Study reported that end of treatment response (ETR) achieved in 80% patients with genotype 3.^28^ This figure was 100% for genotype 1 and 100% for mixed genotypes 5 + 6. In 2.7% patients, treatment had to be discontinued due to side effects of medication and 5.4% patients were lost on follow-up.

## HEPATITIS E

Acute hepatitis due to hepatitis E virus (HEV) is endemic in Bangladesh. Although acute HEV is seen sporadically round the year and outbreaks are common in the monsoon and after floods which this allows sewerage contamination of supply and ground water. However, sporadic outbreaks are also reported.^29^ Genotype 1 HEV is common in Bangladesh.^29^ It has been shown that 58.33% acute viral hepatitis in Bangladesh is due to HEV. The virus is also responsible for 56.52% cases of fulminant hepatic failure (FHF) and 21.7% cases of decompensation of liver cirrhosis.^30^ While HEV is associated with 80% mortality in 3rd trimester of pregnancy, it also remains the leading cause (21.7%) acute insult in patients with another potentially fatal liver disease, acute on chronic liver failure (ACLF).^31^

## DISTRIBUTION OF LIVER DISEASES

In a recent retrospective study, data of 59,227 patients, aged from 15 to 95 years patients from the seven different administrative divisions of Bangladesh between January 2012 and 2013 were analyzed. Although all patients presented at the department of hepatology, 13.2% were diagnosed with liver diseases. Patients with liver diseases were mostly suffering from chronic liver diseases (CLD) (37-69%). Complication of CLD, like hepatic encephalopathy, was less frequent in regions with better healthcare system. Nonviral infections, like liver abscess and biliary ascarisis, were not uncommon. Acute hepatitis was another very common entity and contributed to approximately 20% cases.^32^

### Hepatocellular Carcinoma

Hepatits B virus remains the leading cause of HCC in Bangladesh. Different studies estimate that in this country, 46.9 to 61% HCC is due to HBV. A recent study showed that HBV is responsible for 41% cases of HCC while HCV accounts for 5% and approximately 20.5% likely to be associated with fatty liver diseases. Mean age of HCC patients in Bangladesh is 51 years.^33^

## CONCLUSION

In short, Bangladesh, like our neighbors harbor large HBV and HCV-infected population. Being the third leading cause of deaths of patients admitted in tertiary hospitals of the country,^34^ liver diseases pose significant burden to the country’s economy and healthcare delivery system. Moreover, HBV infection in Bangladesh is relatively unusual, nontreatment friendly and rather aggressive. Studies further demonstrate that considerable numbers of apparently healthy subjects in Bangladesh are unaware of the fact that they are chronically infected by HBV and many of them have already developed progressive liver damage. Local and regional strategies are, therefore, needed for containment and management of HBV infection in our region and our hepatologists, with their limited resources, are currently trying hard to address these issues. This will be further strengthened when organizations, like Miyakawa Memorial Research Foundation (MMRF), will come forward.
